# Pediatric Kidney Biopsy: Clinical Perspectives Based on Survey of Pediatric Nephrologists and Interventional Radiologists

**DOI:** 10.1016/j.xkme.2024.100859

**Published:** 2024-06-18

**Authors:** Nikhil Nair, Charles Varnell, Manish Patel, Jonathan VanGeest, Matt Grinsell, Kathleen Altemose, Sidharth K. Sethi, Rupesh Raina

**Affiliations:** 1 Case Western Reserve University School of Medicine, Cleveland, OH; 2 Division of Nephrology and Hypertension, Cincinnati Children's Hospital Medical Center, Cincinnati, OH; 3 Department of Pediatrics, University of Cincinnati College of Medicine, Cincinnati, OH; 4 Division of Interventional Radiology, Cincinnati Children's Hospital Medical Center, Cincinnati, OH; 5 Department of Radiology, University of Cincinnati College of Medicine, Cincinnati, OH; 6 College of Public Health, Kent State University, Kent, OH; 7 Division of Pediatric Nephrology, Primary Children's Hospital, University of Utah, Salt Lake City, UT; 8 Division of Pediatric Nephrology and Hypertension, Penn State College of Medicine, Penn State Health Children's Hospital, Hershey, PA; 9 Kidney and Renal Transplant Institute, Medanta, The Medicity Hospital, Gurgaon, India; 10 Akron Nephrology Associates/Cleveland Clinic Akron General Medical Center, Akron, OH; 11 Department of Nephrology, Akron Children’s Hospital, Akron, OH

To the Editor:

Many significant renal conditions present with unspecific clinical symptoms such as hematuria, proteinuria and/or anuria and require percutaneous ultrasound-guided kidney biopsy for definitive diagnosis and management. Kidney biopsy adequacy is defined by the quality and quantity of glomeruli able to be collected per pass.^1^ Although this procedure has traditionally been performed by nephrologists, the growth and diversification of interventional radiology (IR) has led to a shift in who performs this procedure, with no agreed on standard for who should perform these procedures.^2^ Almost 70% of recently graduating adult nephrology fellows no longer think that they should be required to demonstrate competence in kidney biopsies as 65%-83% of practicing adult nephrologists do not perform this procedure.^3^^,^^4^ To gauge the thoughts of each specialty, a survey was created to investigate comfort, efficacy, and thoughts on performing kidney biopsies.

The survey was created by the authors representing both specialties to capture relevant feelings and data on the experience of being trained to perform kidney biopsy. All 3 questionnaires were created to ensure face validity and a strong quality of questions without leading or confusing questions. The final questionnaires were sent out to large pediatric nephrology (*pedneph* and *pccrt)* and Society of pediatric interventional radiology (SPIR) listservs.

A total of 280 respondents (pediatric interventional radiologist [PIR] = 29, pediatric nephrologist (PN) = 202, and pediatric nephrology fellow [PNF] = 49) participated in the survey. A statistically significant difference was observed for the performance-related attributes such as confidence about adequately performing a native kidney biopsy (strongly agree, PIR 100% vs PN 51.3% vs PNF 40%; *P* = 0.023); adequately performing a renal transplant biopsy (strongly agree, PIR 100% vs PN 51.3% vs PNF 46.7%; *P* = 0.012); requirement of assistance while performing a kidney biopsy (never, PIR 88.9% vs PN 28.7% vs PNF 3.3%; *P* < 0.001). The reported rate of complications did not differ significantly across the 3 groups (Table 1). The most common complication reported by the respondents were hematoma (33 [41.6%]), hematuria (23 [31.1%]), bleeding (14 [18.9%]), pain (2 [2.7%]), and inadequate specimen (1 [1.4%]). However, the proportion of respondents performing a repeat biopsy was significantly higher among pediatric nephrologists than pediatric interventional radiologists and pediatric nephrology fellows (Table 2). Consistent with the current data, we found pediatric kidney biopsy is safe and consistent to that found in the literature.^5^

**Table 1:** Comparison of the Responses Related to Kidney Biopsy Among the Three Groups

Responses Related to Kidney Biopsy		Pediatric Interventional Radiologist	Pediatric Nephrologist	Pediatric Nephrology Fellow	Total	*P* Value
Perform kidney biopsy at your institute	Yes	11 (100%)	120 (78.9%)	31 (96.9%)	162 (83.1%)	0.012
	No	0 (0%)	32 (21.1%)	1 (3.1%)	33 (16.9%)	
Number of years performing any type of kidney biopsy	1-5	0 (0%)	15 (10.8%)	Not Asked	15 (10%)	0.031
	6-10	6 (54.5%)	23 (16.5%)		29 (19.3%)	
	11-15	2 (18.2%)	29 (20.9%)		31 (20.7%)	
	>15	3 (27.3%)	72 (51.8%)		75 (50%)	
Number of native kidney biopsies performed per year	0	0 (0%)	13 (11%)	0 (0%)	13 (8.3%)	0.018
	1-20	3 (33.3%)	73 (61.9%)	22 (73.3%)	98 (62.4%)	
	21-30	2 (22.2%)	14 (11.9%)	5 (16.7%)	21 (13.4%)	
	31-50	2 (22.2%)	12 (10.2%)	0 (0%)	14 (8.9%)	
	>50	2 (22.2%)	6 (5.1%)	3 (10%)	11 (7%)	
Number of kidney transplant biopsies performed per year	0	0 (0%)	31 (26.3%)	2 (6.7%)	33 (21%)	0.032
	1-20	7 (77.8%)	70 (59.3%)	24 (80%)	101 (64.3%)	
	21-30	1 (11.1%)	13 (11%)	3 (10%)	17 (10.8%)	
	31-50	0 (0%)	4 (3.4%)	1 (3.3%)	5 (3.2%)	
	>50	1 (11.1%)	0 (0%)	0 (0%)	1 (0.6%)	
Type of kidney biopsy performed most frequently	USHB	10 (83.3%)	136 (93.2%)	27 (90%)	173 (92%)	0.207
	Percutaneous based	1 (8.3%)	7 (4.8%)	2 (6.7%)	10 (5.3%)	
	USOB	0 (0%)	0 (0%)	1 (3.3%)	1 (0.5%)	
	CTHB	0 (0%)	1 (0.7%)	0 (0%)	1 (0.5%)	
	Others	1 (8.3%)	2 (1.4%)	0 (0%)	3 (1.6%)	
Type of kidney biopsy performed most frequently by your institution	USHB	10 (83.3%)	Not asked	29 (96.7%)	39 (92.9%)	0.192
	USOB	0 (0%)		1 (3.3%)	1 (2.4%)	
	Percutaneous based	1 (8.3%)		0 (0%)	1 (2.4%)	
	Others	1 (8.3%)		0 (0%)	1 (2.4%)	
Believe that kidney biopsies should be performed by pediatric nephrology	Yes	0 (0%)	95 (81.9%)	Not Asked	95 (76%)	<0.001
	No	9 (100%)	21 (18.1%)		30 (24%)	
The cost of a kidney biopsy at your institute	Do not know the exact cost	3 (50%)	57 (76%)	13 (59.1%)	73 (70.9%)	0.110
	Free for patient	0 (0%)	1 (1.3%)	2 (9.1%)	3 (2.9%)	
	Know the exact cost	3 (50%)	17 (22.7%)	7 (31.8%)	27 (26.2%)	
Cost of a kidney biopsy at your institute (US dollar)	N	3	17	7	27	0.872
	Median (IQR)	400 (190-1,162)	600 (144-2,000)	500 (200-1,600)	500 (195- 1,800)	

Abbreviations: IQR, interquartile range; USHB, ultrasound-guided–hospital based; USOB, ultrasound-guided–office based.

**Table 2:** Comparison of Biopsy-Related Complications, Observation After Biopsy and Repeat Biopsy Among the 3 Groups

		Pediatric Interventional Radiologist	Pediatric Nephrologist	Pediatric Nephrology Fellow	Total	*P* Value
Biopsy-related complications while performing a kidney biopsy	Yes	5 (55.6%)	60 (53.6%)	14 (46.7%)	79 (52.3%)	0.769
	No	4 (44.4%)	52 (46.4%)	16 (53.3%)	72 (47.7%)	
Average number of hours patients are observed after kidney biopsy before discharge	<6	4 (44.4%)	29 (25.2%)	6 (20.7%)	39 (25.5%)	0.533
	6-12	3 (33.3%)	32 (27.8%)	10 (34.5%)	45 (29.4%)	
	>12	2 (22.2%)	54 (47%)	13 (44.8%)	69 (45.1%)	
Performed a repeat biopsy on a patient because of an error while collecting or analyzing the sample the first time	Yes	1 (11.1%)	30 (26.3%)	2 (6.9%)	33 (21.7%)	0.044
	No	8 (88.9%)	84 (73.7%)	27 (93.1%)	119 (78.3%)	

An American Society of Pediatric Nephrology/American Academy of Pediatrics survey in 2013 of pediatric nephrologists surveyed found that 72% conduct kidney biopsies and 81% reported that pediatric nephrologists performed most pediatric kidney biopsies at their respective hospitals.^6^ Those who did not report performing kidney biopsies unanimously reported that IRs performed these procedures. Ten years later that number has increased to 79% of practicing nephrologists and 100% of IRs. IRs were more likely to report feeling comfortable with the procedure and required less assistance compared with nephrologists. As primarily proceduralists, IRs are more comfortable with all procedures overall; however, for pediatric nephrologists, this may be the only procedure they perform.

Concerning training, the specialized procedural aspect of IR residency allows them to be better able to handle complicated cases and management of severe complications.^7^ In most institutions, pediatric IRs perform biopsies considered “more complicated,” including those in which patients are coagulopathic, obese, and/or have difficult transplanted anatomy.^7^ Currently for pediatric nephrologists, there is no requirement or minimum number of biopsies that need to be performed during pediatric fellowship, with expectations varying widely across training programs.^8^ In a recent survey, only a slim majority of program directors feel that kidney biopsies should continue to be taught to pediatric nephrology fellows.^9^ Although it can be argued that an IR may be better trained or more comfortable to perform the biopsy procedure, in theory having the biopsy performed by a nephrologist would allow for the real-time decision-making with regards to tissue adequacy depending on the disease being evaluated.^7^

For either practitioner practicing in low volume settings, avenues for improved and additional training might include attending workshops and simulations. Procedural simulation trainings for kidney biopsies can improve trainee confidence with recent studies finding upward of 94% of fellows being able to procure an adequate yield with post training.^10^ With advances in virtual reality technology, more realistic recreation of ultrasound-guided biopsies and allow for practice of a multitude of techniques.

In any given center, the issue of who performs a kidney biopsy should be decided by local factors and expertise with results recorded and audited in a collaborative manner by all the specialists involved. If the pediatric nephrology field decides that this is an important procedure for the nephrologist to perform, then it will be imperative to gather all stakeholders responsible for pediatric nephrology training to develop clear guidelines and training metrics for kidney biopsies. There may be an opportunity for high quality and effective advanced simulation or virtual reality training, which is becoming more common in medical training to address this gap. Further prospective studies are needed to compare technical success and complication rates amongst these specialties.
